# The Role of Heat Shock Proteins in Insect Stress Response, Immunity, and Climate Adaptation

**DOI:** 10.3390/insects16070741

**Published:** 2025-07-21

**Authors:** Davide Banfi, Tommaso Bianchi, Maristella Mastore, Maurizio Francesco Brivio

**Affiliations:** Laboratory of Applied Entomology and Parasitology, Department of Theoretical and Applied Sciences (DiSTA), University of Insubria, 21100 Varese, Italy; davide.banfi@uninsubria.it (D.B.); tbianchi1@studenti.uninsubria.it (T.B.); maristella.mastore@uninsubria.it (M.M.)

**Keywords:** HSP, insects, abiotic stress, biotic stress, climate change, immunity

## Abstract

Heat shock proteins (HSPs) play a key role in enhancing insect resilience to abiotic and biotic stresses by preserving cellular integrity and modulating immune responses. This review summarizes the main functions of HSPs in insects, including protein stabilization, interaction with antioxidant systems, and involvement in the innate immune response. The expression of HSPs under environmental conditions reflects their evolutionary adaptation to various stressors, including thermal changes, chemical exposure, and pathogens. Future research should focus on the interaction between HSPs and other stress response systems to improve our understanding of insect adaptation. Furthermore, in the context of global climate change, HSPs emerge as a crucial resilience factor and potential biomarkers for environmental monitoring.

## Simple Summary

Heat shock proteins (HSPs) are critical for insect resilience, protecting cellular integrity, and regulating immune responses against abiotic and biotic stresses. This review illustrates the functions of HSPs in insects, including protein stabilization and their role in innate immunity. The expression of HSPs reflects evolutionary adaptations to stressors such as changes in temperature, chemicals, and pathogens. Given the drastic temperature changes associated with global climate change, knowledge about HSPs may become essential to fully understand their role as potential biomarkers for environmental monitoring and pest management.

## 1. Introduction

Insects depend on external environmental conditions to regulate their body temperature, making them highly susceptible to changes in temperature, humidity, or toxicant exposure that can disrupt critical cellular processes. This reliance on ambient temperatures to control metabolism heightens their vulnerability to thermal extremes, which can lead to protein denaturation, enzymatic dysfunction, and loss of membrane integrity [1]. The extensive ecological and taxonomic diversity exhibited by insects, which includes species adapted to an array of habitats and life strategies, makes them exceptionally valuable models for investigating the functions of HSPs across diverse environmental conditions and biological contexts. This diversity is increasingly important as climate change intensifies thermal extremes, amplifying the importance of HSPs for insect survival. Global warming introduces unprecedented pressures, accelerating metabolic rates, increasing dehydration, altering reproductive cycles, and reshaping ecological interactions [2]. Together with anthropogenic stressors like pesticides and habitat fragmentation [3], these factors create a complex stress landscape that tests insect resilience. This review aims to summarize current knowledge on the induction of heat shock proteins (HSPs) under various stressors, elucidate their roles in stress tolerance and immune responses, and underscore their broad relevance in the context of ongoing environmental changes.

**Figure 1:**
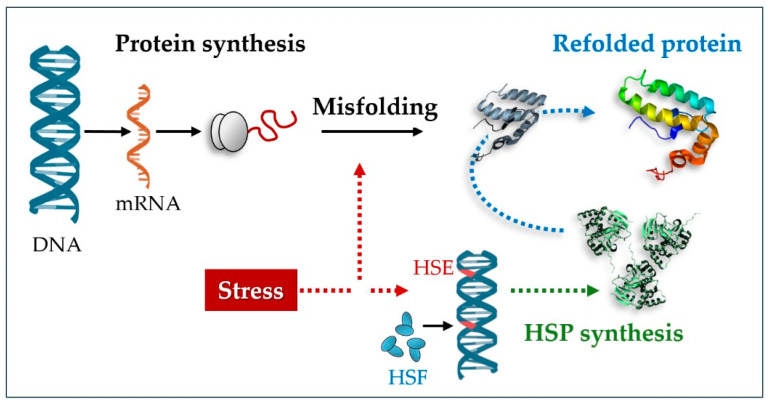
An illustrative scheme of the function of HSPs. Stressors can induce alterations in the molecular architecture of proteins by affecting their functionality; the presence of HSPs controls or restores proper folding while maintaining their functionality.

Heat shock proteins (HSPs), first identified in *Drosophila melanogaster* through heat-induced chromosomal puffing [4,5], serve as key molecular chaperones that maintain protein integrity under such stressors. These proteins, encompassing ATP-independent small HSPs (sHSPs, 12–43 kDa) and ATP-dependent families like HSP60, HSP70, and HSP90, perform specialized roles facilitating the folding of nascent polypeptides, preventing aggregation of denatured proteins during stress, and aiding cellular recovery post-exposure [6,7]. The induction of HSPs is orchestrated by heat shock factors (HSFs), which bind to heat shock elements (HSEs) within promoter regions of target genes. These HSEs are specific DNA sequences recognized by HSFs, functioning as transcriptional activators that upregulate HSP gene expression in response to various stress stimuli, particularly thermal stress [8] (Figure 1).

HSFs, in particular HSF1, are the primary drivers of heat shock protein (HSP) gene transcription [9]. However, HSFs are not the sole regulators of HSP expression since other transcription factors contribute significantly in a context-dependent manner. Feedback loops and crosstalk integrate HSP expression into broader cellular responses. For instance, DREF may be involved in the modulation of Hsp70 expression via DNA replication-related elements during development [10], the GAGA factor enhances chromatin accessibility and directly regulates Hsp70 and Hsp26 through (GA)n sequences [11], and STATs are able to modulate HSP70 and HSP90 gene promoters, playing an important role in HSPs gene activation [12]. Epigenetic mechanisms, including histone acetylation by CBP/p300 [13] and chromatin remodeling by the SWI/SNF homolog BAP [14], further regulate HSP gene accessibility. Thus, while HSF1 is central, a complex network of transcription factors and epigenetic regulators ensures precise, context-specific HSP expression.

The induction of HSPs is not confined to stress exposure [15]; instead, they can be constitutively expressed at basal levels to maintain protein homeostasis. Many of these factors are also present during normal physiological states, where they function as normal regulators of protein activity. HSPs could also be encoded by constitutive gene pathways, known as Heat Shock Cognates (HSC70 family), which are expressed under non-stress conditions and assist in post-translational protein folding and membrane integration. In contrast, inducible HSPs, e.g., HSP27, HSP70, HSP90, produced in response to cellular stress, help fold and stabilize partially denatured proteins, thereby preserving proteostasis. For example, in *Bombyx mori*, *Bemisia tabaci*, and various *Drosophila* species, both inducible (HSP70s) and constitutive (HSC70s) forms of molecular chaperones are present. Their expression patterns vary depending on developmental stage and tissue type across different body regions [16,17,18,19].

Recent studies suggest that HSPs also play a role beyond stress response, contributing significantly to key immunological and developmental processes. For instance, several investigations in *Aspongopus chinensis* have identified 25 HSP genes with distinct expression patterns between diapause and non-diapause stages; notably, the significant downregulation of HSP70-5 during diapause highlights a critical link between endocrine regulation and immune readiness [20]. Moreover, evidence from *Bombyx mori* indicates that 16 sHSPs exhibit tissue-specific expression with differential peaks in the midgut during feeding and in the gonads during pupation, suggesting developmental roles that extend beyond immediate stress responses [21]. By changing the normal physiological level, under abiotic (thermal or chemical) or biotic stress conditions, HSP90 increases significantly, enabling insects to withstand environmental challenges.

**Figure 2:**
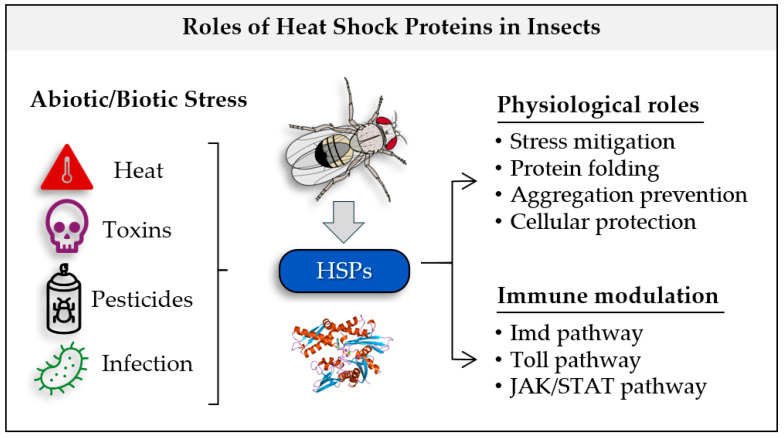
Physiological and immunological role of insects’ HSPs.

HSPs play a crucial role in immune responses, integrating into the sophisticated signaling networks that underpin insect immunity. Three primary immune pathways, the IMD, Toll, and Jak/STAT pathways (Figure 2) govern the recognition of pathogens and the orchestration of immune defenses [22]. The IMD pathway, targeting Gram-negative bacteria, activates a cascade involving proteins such as IMD, FADD, and Dredd, leading to the transcription of antimicrobial peptides (AMPs) like defensins and cecropins [23,24]. The Toll pathway specializes in defense against Gram-positive bacteria and fungi [22], employing receptors like Toll and Spätzle to initiate downstream signaling through MyD88, Tube, and Pelle, culminating in the production of AMPs via transcription factors Dorsal and Dif [25]. Meanwhile, the Jak/STAT pathway is essential for the innate immune system, playing a crucial role in not only defending insects against bacterial, fungal, and viral pathogens but also in tissue repair, relying on cytokines or stress signals to activate Jak kinases and STAT transcription factors that regulate genes encoding AMPs and stress-response proteins [26,27].

Emerging research highlights the potential interactions between HSPs and these immune pathways. HSPs, such as HSP70 and HSP90, may modulate immune pathway activity by stabilizing key signaling proteins, enhancing transcription factor activation, or facilitating the rapid turnover of immune components. These interactions suggest that HSPs contribute not only to stress tolerance but also to immune resilience, underscoring their dual protective role in the face of environmental and biological challenges.

## 2. Thermal Stress and Heat Shock Proteins

Insects are routinely exposed to extreme thermal conditions that can disrupt protein structure, elevate oxidative stress, and impair cellular homeostasis. With climate change intensifying, these challenges are becoming more severe and rising global temperatures with increasingly uneven weather patterns exacerbate thermal stress, impacting insect survival, reproduction, and ecosystem functions; in this context, HSPs are pivotal in mounting rapid and coordinated defenses against such abiotic challenges. For instance, studies on the locust bean moth, *Ectomyelois ceratoniae*, demonstrate that exposure to extreme temperatures, whether intense heat or severe cold, stimulates the production of these proteins. Interestingly, HSP70 is mainly expressed during cold stress, whereas HSP90 shows higher expression levels under heat, indicating their specialized roles in handling distinct thermal challenges in this insect [28]. In the bumblebee *Bombus terrestris*, thermal stress at temperatures of 9 °C or 38 °C leads to a three-fold increase in HSC (HSP70 family) and its co-chaperone Aha. Remarkably, this upregulation occurs without compromising immune function, suggesting that HSP expression can be precisely modulated to address both thermal and immunological demands [29]. The nocturnal moth *Spodoptera litura*, part of the family Noctuidae, exhibits broad-spectrum thermal protection through the induction of SlHSP20.4 under both heat (42 °C) and cold (5 °C) stresses. This response likely operates under the regulation of distinct isoforms of HSFs, enabling the organism to withstand diverse stress conditions [30]. Similarly, in the bloodsucking bug *Rhodnius prolixus*, HSP70 expression significantly increases at both 0 °C and 40 °C, supporting vital physiological functions such as digestion and molting. RNAi-mediated silencing of HSP70 in this species results in a 60% reduction in survival, underscoring the indispensability of this protein in thermal tolerance [31]. In contrast, the brown planthopper *Nilaparvata lugens* exhibits a biphasic response to thermal stress, HSP70 levels remain stable between 28 °C and 40 °C, dropping significantly during cold stress at 5 °C, only to rebound at 25 °C; this pattern suggests a sophisticated adaptive mechanism that may involve post-translational modifications and interactions with co-chaperones like HSP40. Furthermore, this response to temperature changes seems to correlate with increased resistance to insecticides such as imidacloprid [32].

Specifically considering heat shock-induced stress, in the aquatic chironomid *Prodiamesa olivacea*, exposure to 35 °C results in a dramatic 128-fold increase in HSP70 expression; this remarkable upregulation stabilizes denatured proteins, making HSP70 a sensitive biomarker for thermal stress in freshwater habitats, where increased temperatures exacerbate oxygen depletion and metabolic demands [33]. Similarly, in larvae of *Glyphodes pyloalis*, heat stress at 40 °C induces multiple HSPs that act synergistically with antioxidant enzymes, such as superoxide dismutase (SOD) and catalase, to counteract oxidative damage [34]. Prolonged exposure to 40 °C in this lepidopteran species significantly upregulates GpHSP71.3 and GpHSP82.4; indeed, following RNAi-mediated silencing of GpHSP82.4, mortality increases by 70%, underscoring its critical protective function under heat stress [35].

In *D. melanogaster*, heat stress at 37 °C rapidly induces HSP70 in a tissue-specific manner, with peak expression within two hours. This response also exhibits significant sex-specific differences, highlighting the fine regulation of thermotolerance mechanisms [36,37]. Moreover, in the springtail *Orchesella cincta*, delayed but sustained HSP70 induction correlates strongly with survival during gradual temperature shifts, confirming the adaptive advantage of prolonged HSP expression under slowly changing environmental conditions [38]. Studies on pest species further elucidate the multifaceted roles of HSPs in thermal stress adaptation. In *Mamestra brassicae*, pre-exposure to 37 °C increases the expression of HSP70, HSP90, and HSP60 by five- to eight-fold, enhancing fungal infection survival by 40% through improved phagocytosis [39]. In *Aedes aegypti*, acute heat stress dramatically upregulates small HSPs, emphasizing the species’ sensitivity to elevated temperatures [40]. Likewise, in *Grapholita molesta*, pupal exposure to 38 °C for four hours enhances HSP70 and HSP21 expression by 15-fold, boosting adult survival by 25% and reproductive output by 30%, a hormetic effect that highlights the adaptive benefits of HSPs under heat stress [41]. In *Galleria mellonella*, heat shock at 40 °C not only increases the abundance of antimicrobial peptides such as cecropin by four-fold, enhancing survival by 50%, but also boosts antifungal activity by 70% when combined with short-term heat shocks [42,43]. Similarly, in *Agasicles hygrophila*, the heat shock factor AhHSF regulates the transcription of AhHSP70 and AhHSP21 between 36 °C and 39 °C, with RNAi silencing of AhHSF reducing survival by 60% [44]. Finally, in *Spodoptera exigua*, HSPs such as SexHSP74 and SexHSP83 play distinct roles in short- and long-term recovery from heat stress [45]. Heat stress also influences the effects of pesticides; for instance, in *Anopheles sinensis*, AsHSP90AB, upregulated eight-fold under heat stress, interacts with cytochrome P450 enzymes to confer pyrethroid resistance [46]. High levels of HSP expression following thermal shock result from the effectiveness of their promoter. For instance, in *Aedes albopictus*, the use of a *Drosophila* HSP70 promoter increases transfection efficiency ten-fold at 41 °C, demonstrating the value of HSP regulatory elements in genetic research [47]. HSPs are also involved in the regulation of hormonal interactions after heat shock. For example, in *Helicoverpa armigera*, HSP90 phosphorylation mediated by protein kinase C facilitates hormonal interactions between 20-hydroxyecdysone and juvenile hormone signaling under heat stress [48].

Considering only low temperatures, studies in *D. melanogaster* have revealed that under cold stress conditions (0 °C), heat shock proteins (HSPs) function as damage-associated molecular patterns (DAMPs), activating the JAK/STAT immune pathway [49]. Similarly, research on *Ostrinia furnacalis* demonstrates that exposure to 8 °C results in six-fold upregulation of small HSPs and HSP90, alongside immune genes. This finding underscores their dual role in facilitating cold adaptation and promoting immune responses [50]. Furthermore, investigations into *Pyrrhocoris apterus* highlight the critical involvement of HSP70 in recovery from chilling injuries at 0 °C. RNAi-mediated knockdown of HSP70 significantly impairs this recovery process, confirming its essential role in cold stress tolerance [51].

## 3. Dehydration Stress and Heat Shock Proteins

Climate change intensifies droughts worldwide, driven by rising global temperatures and altered precipitation patterns, all of which reduce water availability. These environmental shifts severely affect insect biodiversity, threatening the essential roles insects play in ecosystems such as pollination, population control, and decomposition. HSPs are recognized as critical molecular tools in insect responses to dehydration and anhydrobiosis. Studies on the Antarctic midge *Belgica antarctica* reveal that HSPs (sHSP, HSP70, HSP90) are rapidly upregulated under dehydration and overhydration, helping maintain cellular homeostasis [52]. The sleeping chironomid *Polypedilum vanderplanki* undergoes anhydrobiosis, a survival mechanism in which HSPs such as Pv-HSP90, Pv-HSP70, Pv-HSC70, Pv-HSP60, Pv-HSP20, and Pv-p23, along with Pv-HSF1, are highly expressed during desiccation. This demonstrates their role in protecting cellular structures and metabolic functions [53]. Additional research, including RNAi studies on mosquitoes (*Aedes aegypti*, *Anopheles gambiae*, and *Culex pipiens*), highlights species-specific variations in HSP expression during dehydration, underscoring their role in water balance [54]. In bed bugs (*Cimex lectularius*), HSP70 and HSP90 are critical for managing dehydration stress, with HSP90 playing a dominant role during recovery [55]. Collectively, these findings underline the importance of HSPs in enabling insect resilience to extreme dehydration, providing key insights into adaptations crucial for survival in changing environments.

## 4. Biotic Stresses and Heat Shock Proteins

HSPs are critical for insect survival because, in addition to maintaining cellular proteostasis, they orchestrate complex immune responses against various pathogenic challenges. Indeed, studies in insects indicate that HSPs play an essential role in immune defense mechanisms [56]; for instance, their expression may be significantly affected by pathogen exposure. In the Chinese oak tussar moth *Antheraea pernyi*, the expression of sHSP21 (*ApsHSP21*) was analyzed in immune tissues using qRT-PCR following different pathogen challenges, including lipopolysaccharide (LPS), peptidoglycan (PGN), glucan, and nucleopolyhedrovirus (NPV). The results showed that *ApsHSP21* expression varied depending on the infecting pathogen. In response to LPS, its expression significantly increased, reaching peak levels at 6 and 12 h after injection. PGN exposure led to an earlier peak at 3 h, with glucan treatment also causing a similar early peak at 3 h post-injection. When challenged with NPV, *ApsHSP21* exhibited a substantial increase, peaking at 36 h post-injection. These findings indicate that sHSP21 plays a dynamic role in immune defense, responding differently depending on the nature of the pathogen [57]. Complementary research on the ApsHSP21.4 isoform reveals its capacity to modulate immune-related gene expression after NPV challenge, with eicosanoid signaling mediated by lipid-derived molecules, such as prostaglandins and leukotrienes, serving as a pivotal regulatory mechanism in this crosstalk [58]. In the red flour beetle *Tribolium castaneum*, exposure to LPS elicits coordinated upregulation of HSPs alongside immune effectors, including defensins and thaumatins, the latter of which disrupt microbial membranes and may have evolved through horizontal gene transfer to enhance antifungal protection [59].

Further complexity is evident in *D. melanogaster*, wherein exposure to viral agents causes context-dependent modulation of heat shock protein (HSP) expression. For example, infection with Drosophila C virus leads to upregulation of HSP levels, a response that may result from differential activation or targeting of HSFs. Viral infections, therefore, appear to act as modulators of HSP synthesis. In cases of upregulation, this response may facilitate viral replication and spread through HSP-mediated assembly of molecular complexes such as viral replicases [60,61,62]. Consistently, inhibition of HSPs has been shown to impair viral replication by disrupting virus-HSP interactions [63]. In contrast, infection of *D. melanogaster* cells with cricket paralysis virus (CrPV) has been reported to suppress HSP expression by approximately 50% [64].

Another study conducted by Zhang and colleagues investigates the interaction between destruxins (DA) and heat shock proteins (HSPs) in silkworm cells; DA induces the up-regulation of Bm*HSP70-3*, Bm*HSP75*, Bm*HSP83*, and Bm*HSCP* genes. Interestingly, the higher expression levels of the HSP70 family (Bm*HSP70-3* and Bm*HSCP*) under DA stress suggest a stronger response compared to the HSP90 family (Bm*HSP75* and Bm*HSP83*) [65]. Another organism widely used in research in this field is *G. mellonella*; for instance, Wojda and colleagues infected this insect with *Bacillus thuringiensis* [66]. The infection induces a five-fold increase in HSP90 expression. More interestingly, the subsequent application of the HSP90 binding compound DMAG-17 results in higher antimicrobial defense due to the enhanced production of antimicrobial peptides (AMPs), improving larval survival. The binding of 17-DMAG to HSP90 disrupts its interaction with associated chaperone proteins, including HSF-1. The latter is a transcription factor that becomes active under heat shock or stress conditions, dissociating from its inactive complex with HSP90 to enable the transcription of stress-related genes. This process is mimicked by 17-DMAG, as it induces the dissociation of Hsf-1 from HSP90 [66].

Regarding biotic stresses caused by organisms other than bacteria, infection with the nematode *Steinernema carpocapsae* deprived of its cuticle modulates HSP90 expression by four-fold, while the untreated and dead nematodes lead respectively to slightly increased and unchanged HSP90 expression, highlighting the direct involvement of HSPs in nematode-induced biotic stress [67]. Another study conducted on *G. mellonella* larvae investigated the role of several HSPs’ responses after infection by the fungus *Conidiobolus coronatus* [68]. HSP60 and HSP90 were prevalent in healthy larvae, while HSP70 and HSP27 were present in trace amounts; upon infection, the levels of HSP60 and HSP27 increased at both 24 h and 48 h, while that of HSP90 increased only at 48 h; no changes were observed in HSP70 levels. These findings highlight the specific roles of HSPs in insects during fungal infection, offering insights into the interplay between insect immunity and pathogens.

Together, these findings highlight the pivotal role of HSPs as adaptable molecular chaperones, seamlessly integrating innate immune signaling with cellular repair mechanisms. This enables insects to thrive in pathogen-rich environments. HSPs are essential for insect survival, ensuring cellular stability while driving adaptive immune responses to diverse pathogens. Research across various insect models has revealed the dynamic and context-dependent expression of HSPs, emphasizing their critical flexibility in addressing specific immune challenges. These studies underscore the importance of HSPs in shaping insect immune defenses, offering profound insights for fundamental research and practical advancements in pest management and immunity studies.

The following Table 1 provides a summary of the stressors and roles of the main HSPs.

## 5. Conclusions

In summary, our mini review underscores the integral role of HSPs in enhancing insect resilience against both abiotic and biotic stressors. The literature cited in this article clearly outlines the multifaceted role of HSPs in physiological control in response to stress. By stabilizing proteins, coordinating with antioxidant defenses, and engaging with various cellular stress response mechanisms, HSPs preserve cellular integrity and enhance resilience against environmental fluctuations.

In the context of global climate change, their roles are increasingly significant, offering insights into adaptive mechanisms that may inform broader strategies for understanding biological resilience. HSPs not only maintain cellular integrity by facilitating protein folding and repair but also actively modulate immune responses through pathways such as Toll/NF-κB and integrin-mediated phagocytosis. The remarkable plasticity in their expression, varying across developmental stages, tissues, and environmental conditions, demonstrates a finely tuned system evolved to withstand thermal extremes, chemical exposures, and pathogen attacks. Furthermore, the dual role of HSPs as protectors of cellular homeostasis and as indicators of environmental change positions them as promising biomarkers for assessing ecosystem health in the context of global climate change. Their potential application in pest management, particularly through RNA interference strategies, highlights the translational relevance of these proteins in developing sustainable control measures while minimizing ecological disruption. Future research is critical to further investigate the complex interactions between HSP networks and other stress response systems, including co-chaperone interactions and cross-tolerance mechanisms. These studies are essential for improving our ability to predict insect responses in the context of increasingly variable climatic conditions, as well as for optimizing strategies that concurrently promote agricultural productivity and biodiversity conservation.

## Figures and Tables

**Table 1:** Stressors and functions of major HSPs.

HSP	Stressors	Functions
HSP 90	Heat and cold Dehydration Rehydration Insecticides Toxins Microorganisms Parasites Starvation	Enhances survival under thermal stress, infections, and insecticide challenges Facilitates protein folding of misfolded proteins during stress, preventing aggregation Prevents aggregation of denatured proteins Stabilizes proteins during stress responses Supports proteostasis under water stress Stabilizes signaling kinases and stress-signal transduction pathways under stress Modulates host-pathogen interactions
HSP 60/70	Heat and cold Dehydration Rehydration Insecticides Toxins Microorganisms Parasites	Enhances survival under thermal stress, infections, and insecticide challenges Facilitates protein folding of misfolded proteins during stress, preventing aggregation Stabilizes proteins under stress conditions Prevents aggregation of denatured proteins Improves thermotolerance and aids recovery after stresses Supports water-loss tolerance, aiding survival after dehydration and anhydrobiosis Promotes stress-signal transduction Exhibits anti-apoptosis effects Play a role in immune regulation
HSPs small	Heat and cold Dehydration Rehydration Microorganisms Virus	Enhances survival under thermal stress and infections Facilitates protein folding of misfolded proteins during stress, preventing aggregation Stabilizes proteins under stress conditions Protects against oxidative stress Promotes innate immune signaling and modulates immune-related genes Stabilizes the cytoskeleton
Detailed information on the HSPs summarized in this table can be found in supplementary material		

## Data Availability

No new data were created or analyzed in this study. Data sharing is not applicable to this article.

## Supplementary Materials

The following supporting information can be downloaded at https://www.mdpi.com/article/10.3390/insects16070741/s1. Table S1: HSP full table.
